# Nanoparticles and Nanomaterials as Plant Biostimulants

**DOI:** 10.3390/ijms20010162

**Published:** 2019-01-04

**Authors:** Antonio Juárez-Maldonado, Hortensia Ortega-Ortíz, América Berenice Morales-Díaz, Susana González-Morales, Álvaro Morelos-Moreno, Marcelino Cabrera-De la Fuente, Alberto Sandoval-Rangel, Gregorio Cadenas-Pliego, Adalberto Benavides-Mendoza

**Affiliations:** 1 Botánica, Universidad Autónoma Agraria Antonio Narro, Saltillo 25315, Mexico; juma841025@gmail.com; 2 Materiales Avanzados, Centro de Investigación en Química Aplicada, Saltillo 25294, Mexico; hortensia.ortega@ciqa.edu.mx; 3 Robótica y Manufactura Avanzada, Centro de Investigación y de Estudios Avanzados Unidad Saltillo, Ramos Arizpe 25900, Mexico; america.morales@cinvestav.edu.mx; 4 CONACYT-Universidad Autónoma Agraria Antonio Narro, Saltillo 25315, Mexico; qfb_sgm@hotmail.com (S.G.-M.); alvarinho001@gmail.com (Á.M.-M.); 5 Horticultura, Universidad Autónoma Agraria Antonio Narro, Saltillo 25315, Mexico; cafum7@hotmail.com (M.C.-D.l.F.); asandovalr16@gmail.com (A.S.-R.); 6 Síntesis de Polímeros, Centro de Investigación en Química Aplicada, Saltillo 25294, Mexico; gregorio.cadenas@ciqa.edu.mx

**Keywords:** biostimulation, stress tolerance, elicitors, corona, hormesis, nutritional quality, growth promoters

## Abstract

Biostimulants are materials that when applied in small amounts are capable of promoting plant growth. Nanoparticles (NPs) and nanomaterials (NMs) can be considered as biostimulants since, in specific ranges of concentration, generally in small levels, they increase plant growth. Pristine NPs and NMs have a high density of surface charges capable of unspecific interactions with the surface charges of the cell walls and membranes of plant cells. In the same way, functionalized NPs and NMs, and the NPs and NMs with a corona formed after the exposition to natural fluids such as water, soil solution, or the interior of organisms, present a high density of surface charges that interact with specific charged groups in cell surfaces. The magnitude of the interaction will depend on the materials adhered to the corona, but high-density charges located in a small volume cause an intense interaction capable of disturbing the density of surface charges of cell walls and membranes. The electrostatic disturbance can have an impact on the electrical potentials of the outer and inner surfaces, as well as on the transmembrane electrical potential, modifying the activity of the integral proteins of the membranes. The extension of the cellular response can range from biostimulation to cell death and will depend on the concentration, size, and the characteristics of the corona.

## 1. Introduction

Biostimulation of an organism is a phenomenon of modification in metabolic processes that allow the more efficient use of environmental resources, more significant growth or yield, and more tolerance to adverse environmental factors. Biostimulation has been described as a general biological phenomena dependent of the interactions between cell molecular structures and external impulses or stimuli [1]. After the application of a biostimulating agent, a sequence of events of perception, transduction, signaling, effector action, and modification in gene expression, metabolism or cellular (and organismal) characters occurs. Environmental agents that cause biostimulation in living organisms can be physical, chemical, or biological [2,3]. In cultivated plants, biostimulation has been defined as the result of applying substances or microorganisms that do not directly add essential elements, known plant hormones, or molecules with suppressive effect of diseases [4,5].

Nanoparticles of metals, semimetals, and non-metals (NPs) and nanomaterials (NMs) of C (such as carbon nanotubes), organic (such as chitosan) and other materials such as nanoclays are materials with at least one dimension less than 100 nm. This small size gives rise to properties different from those exhibited by the bulk material of the same composition. These new properties provide the material with an added value that has multiple applications in automotive, energy, pharmaceutical, medical, and agricultural industries, among others [6].

In the present manuscript it is proposed that due to the physicochemical characteristics of the NPs and NMs, as well as the responses induced in the plants, NPs and NMs could be considered in the group of compounds useful for crops known as biostimulants. The capacity of these materials, applied in generally small amounts and by foliar spray or in nutrient solutions, has been described to modify the composition and nutraceutical quality of food crops, as well as their tolerance to stress [7,8,9]. The purpose of the proposal is that, at a time when the legislation on agricultural biostimulants is being discussed [4,5], it is perhaps an appropriate time for the consideration of NPs and NMs to be included as a kind of biostimulant, taking into account the enormous potential utility for the agricultural sector. In this context, the effectiveness of NPs and NMs applied in minimal quantities should be highlighted. The use in low concentrations is recommended because it decreases the risk of a plant’s toxicity [10,11] and, in the case of NPs of metals such as Fe, Mn, and Cu, the low amounts applied may decrease the transfer to the soils and food chain, in comparison with fertilizer bulk materials [12].

Apparently, biostimulation by NPs and NMs occurs in two phases. The first is of a physicochemical nature through the energy and surface charges of the NPs and NMs, which are associated with the surfaces of walls and cell membranes modifying the activity of receptors, transporters, and other proteins, also modifying the transport of metabolites and ions to cells [13]. The second phase occurs through biochemical processes that respond to the release of the chemical elements from the NP or NM (for example Fe^3+^ from Fe NPs or carbon compounds from NPs of chitosan) and that would be analogous to the responses to metal ions or organic molecules in their bulk form [14,15]. The first phase, the interaction of surfaces, in the case of a physicochemical process, can be independent from the composition, this is why it is possible that practically any type of inorganic (Cu, CuO, silica) or organic (polystyrene, chitosan) NP or NM in specific concentration ranges has this kind of biostimulating capacity. Indeed, it has been proposed that the surface area of the NPs and NMs, instead of the concentration, be used as a predictor of the potential biological impact [16].

In addition to the Introduction, the manuscript has three main sections. In Section 2, the subject of the superficial charges of the cells is included. Section 3 consists of the explanation about the surface charges of the NPs and NMs and the interaction of the surface charges of cells and NPs and NMs; explaining how the interaction process can cause biostimulation. Section 4 includes examples of the biostimulation process.

## 2. The Charges of the Cell’s Surface

**Figure 1:**
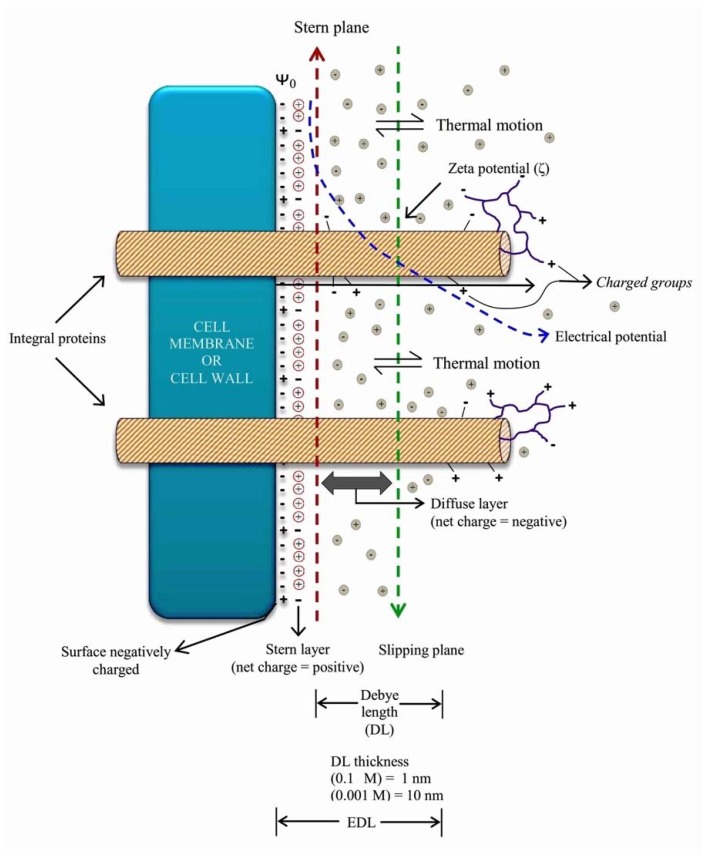
Schematic representation of the electric double layer (EDL) as a dynamic structure on the surface of a charged cell wall or membrane of a living cell, nanoparticle (NP) or a nanomaterial (NM) when it is exposed to a fluid. The term EDL refers to two parallel layers of charge on the surface. The Stern layer (with a positive net charge), consists of ions adsorbed onto the surface due to chemical interactions. The diffuse layer (with a negative net charge) is composed of ions attracted to the surface charges of the Stern layer via the Coulomb force. The diffuse layer is made of free ions that move in the fluid under the influence of electric attraction and thermal motion. The Debye length is the thickness of the mobile ions of the EDL and marks the distance under the influence of the electric potential of the surface. The zeta potential is the electrical potential at the slipping plane. The volume contained under the slipping plane shows tangential molecular motion with respect to the surface. In diluted solutions (0.001 M) the Debye length is about 10 nm but decreases with the ionic strength (1 nm with 0.1 M). In plants, the Debye length is within 1–2 nm, because in biological fluids the ionic strength is often about 0.15 M [20]. Since the transmembrane domains of integral proteins can protrude from 2 to 7 nm [21], groups with positive and negative charges of proteins and recognition sites or receptors are located outside the EDL. On the other hand, the peripheral proteins associated with integral proteins are located even further away from the EDL.

The walls and cell membranes, the latter through the plasmodesmata [17], are in contact with the apoplast, where there are abundant organic and inorganic chemical species with charge. Several chemical interactions occur at the wall-membrane-apoplast interface, such as acid/base dissociations, ionization of functional groups, ion adsorption or other species charged on surfaces, as well as the partial dissolution of some of the structural components of the membranes and cell walls. The intensity and final balance of these interactions depend on the pH, ionic strength, oxidation-reduction potential, and other dynamic properties of the extracellular medium. When reaching a point of dynamic equilibrium between the cell surfaces and the fluid medium, the interface acquires a net charge negative since the number of positive charges at the interface is less than that of negative charges [18]. It modifies, by attraction/repulsion, the random distribution created by the thermal movement of the ions in the solution of the apoplast. The result is a dynamic electric double layer (EDL) in the region near the surface of the membrane [19] (Figure 1).

The net negative charge of cell surfaces depends on the sum of negative and positive charges at the interface with the external environment. The net negative charge occurs since the number of positive charges at the interface is less than that of negative charges. The net negative charge of cell surfaces determines the interaction with the ions in solution both in the EDL of the external side (apoplast) and in the inner side of the cell membrane. The ions in solution in the apoplast are available at the cell interface through diffusion, and their concentration and activity depend on the final balance of charges at the interface [18]. On the other hand, the number of charges per unit area (charge density), determines the electrical potential of the surface of the membranes represented by ψ_0_ (Figure 1) as well as the transmembrane potential that maintains the structure and functionality of the integral proteins [18,19].

Additionally, to the primary surface charges of the membrane that fundamentally make up the EDL, there are negative and positive charges resulting from the presence of integral and peripheral proteins (receptors, channels, response proteins, among others), and of the lateral flow of protons associated with proton pumps [22]. The same occurs in the cell wall, where metabolism-related and response-related proteins determine the presence of negative and positive free charges, in addition to the surface charges of structural biomolecules that make up the EDL (Figure 1). These free charges of a membrane´s or cell wall´s proteins, can be found outside the main volume of influence of the EDL. They offer molecular interplay sites with a mixture of negative and positive charges to biomolecules, polymers, xenotoxics, NPs, and NMs. The interactions can be of the non-specific electrostatic type, like those that supposedly occur with NPs and NMs, or recognition interactions with native receptors of surface proteins [23], both interactions being able to induce plant biostimulation [24].

## 3. The Surface Charges of Nanoparticles (NPs) and Nanomaterials (NMs) and Its Interaction with Cell Surface Charges

While several properties of NPs and NMs such as surface charge, surface energy, size, shape, roughness, porosity, hydrophobicity, and hydrophilicity have an impact on the interaction with cell surfaces [20,25], the emphasis in this manuscript will be focused on the charges and the charges-related energy surface of the materials. The reason is that the above enumerated set of properties of the NPs and NMs seem to modify the interactions with the cells increasing their internment in the cells [26], but it is the interaction between the surface charges of NPs–NMs–cells that initially trigger changes in metabolism and gene expression in cells [27].

Like any other material, the NPs and NMs have superficial free energy derived from the exposure of the electrons at the interface of the material and the surrounding medium. The difference with the bulk material is that the NPs and NMs, due to their high surface/volume ratio, have a much higher amount of surface free energy, which mainly explains the higher reactivity of the materials in the nanometric form [16,28,29]. This high level of superficial free energy supposes an entropy imbalance that tends to diminish spontaneously, through the interaction with other molecules or forming aggregates of NPs that precipitate. In both cases the surface exposed at the interface with the medium is decreased [30].

When the NPs or NMs are placed in a natural environment such as water, soil, epidermis, or the internal fluids of plants, they adsorb organic matter components or biomolecules (mainly proteins and peptides) from the environment, forming a corona. The composition of the corona depends initially on the most abundant biomolecules (soft corona), but over time adopts a particular composition that probably has the lowest free energy (hard corona), without necessarily including the most abundant biomolecules of the medium [31]. The corona formation is a mechanism for decreasing the surface free energy of NPS or NMs, so it happens spontaneously, regardless of whether the character of the pristine NP is cationic, anionic, or neutral [23].

The formation of the corona on a NP or NM occurs by the action of biomolecules or organic molecules that also have surface charges, forming molecular monolayers when their molecular weight is high (such as albumin) or multilayers of molecules when the molecular weight is low (such as lysozyme) [32]. The coronas of the NPs or NMs have a high density of surface charges and therefore high free energy, since it is concentrated in a small volume with large exposure area. A direct relationship has been reported between the number of proteins present in the corona and the surface charge density of the NP or NM [33,34].

**Figure 2:**
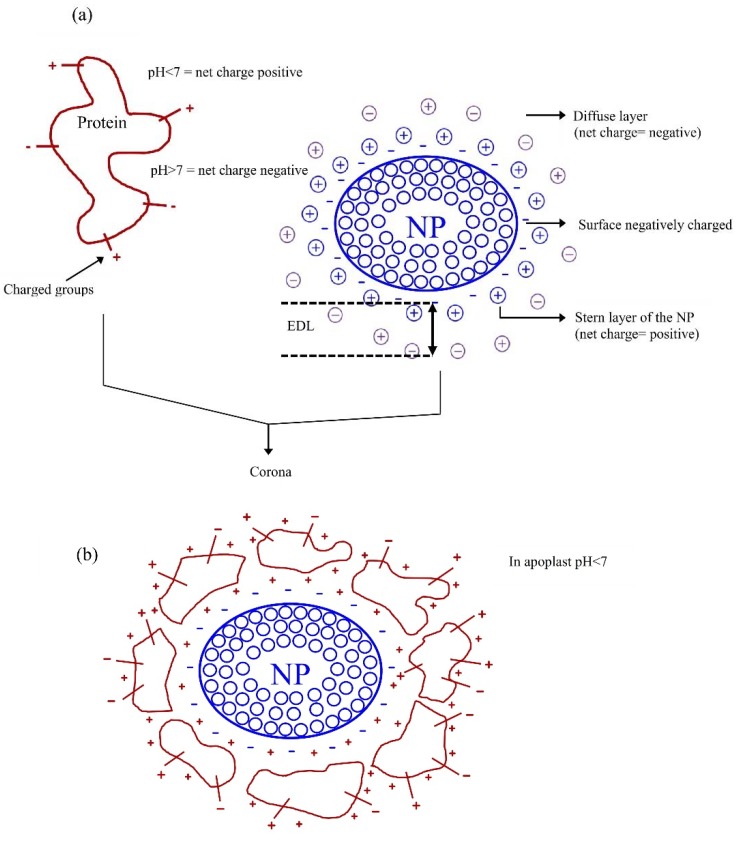
Graphical representation of formation of NP and NM corona in natural fluids. In (**a**) (left side) the net surface charge of the protein is positive at pH < 7 (with a negatively charged Stern layer). In (**a**) (right side) the pristine nanoparticles (NPs) shows a surface negative charge and a positive charged Stern layer. In (**b**) as a result of the diffuse layers of proteins and NPs having opposite charges, the electrostatic interactions that give rise to the corona occur.

The surface charge of pristine state of NPs and NMs in a natural environment is commonly negative, with a positive Stern layer and a negative diffuse layer, forming its EDL with a very high density of charges (Figure 2a) [28]. In the laboratory or during the industrial process, the original surface with negative net charge can be modified by functionalizing NPs or NMs, taking a positive, negative, or neutral character [35].

The formation of the corona depends on the chemical characteristics of the environment and on the surface charge of the chemicals used to functionalize the NPs and NMs [23]. In particular, when a corona is formed by interacting the NPs with natural fluids (with pH values around or less than 7), the cover formed by proteins or peptides will have a positive surface charge, with a negative Stern layer and a positive diffuse layer, resulting in a corona with an EDL of positive net surface charge [36] (Figure 2b), whose magnitude will change with the values of pH and ionic strength of the solution. The corona composition is modified as long as NPs are transferred from one medium to another, for example, from an abiotic to a biotic environment (and vice versa) or from an extracellular to an intracellular environment [37]. Even in structurally and chemically identical NMs and NPs, the structure of the different proteins in the corona determine the cellular receptors and components with which the NPs interact [23] since the NPs express functional epitopes depending on the type of corona [38].

The positive surface charge of the proteins in the corona have a negative Stern layer (contrary to the positive Stern layer of the cell surfaces), which probably will dictate most of the interactions with both the ions and biomolecules of the apoplast and the initial attraction with the EDL of the cell surfaces. The initial interactions do not prohibit the interaction with anionic or cationic components of cell walls or membranes through positive and negative charge groups located on the surface of the corona [23] or in the core of the NP or NM that is not entirely covered by the corona [39].

The composition of the corona dictates to a large extent the capacity of binding with the receptor sites of the cell wall and cell membrane [40]. The interaction between the corona of NPs and NMs and cell surfaces is a result of the decrease in free surface energy of the corona when attaching to the components of the cell wall or membrane [20].

In the proteins that form the corona, the net surface charge that interacts with the EDL of NPs and NMs depends on the isoelectric point of each protein. In eukaryotes, the isoelectric point of proteins varies in a pH range of 6 to 8 [41], which partially explains the dynamic character of the corona and its different composition in different biological media, since the pH can vary with values from 5 to 8 in the different cell compartments [42]. The different composition of the coronas of NPs creates a specific chemical identity, constituting a factor that adds complexity to the impact of NPs in plants. According to the characteristics of the corona, the same type of NPs can produce different cellular responses, from biostimulation to toxicity [40].

In addition to the above, the surface charges of metallic NPs form a very reactive electronic plasma that, in addition to interacting with the biomolecules of the medium, also do so with photons, creating plasmons which under certain conditions trigger cellular responses [43].

Moving now to the topic of cell wall and membrane surface charges, the amount of electric charges per unit area is the density of charges (σ = q/A; q = charge in Coulombs (C), A = area). The number of binding sites with a negative charge on the surface of a membrane was established with a value of 0.3074 μmol m^−2^, while the number of neutral sites (phospholipids) was determined with a value of 2.4 μmol m^−2^. The density of charges on the surface of a plant cell membrane was estimated with a value of σ_0_ = −30 mC m^−2^ [18].

The density of charges modifies the cellular behavior against ions by changing the electrical potential of the surface of the membranes (ψ_0_) as well as the transmembrane potential that keeps the ion transport channels and other integral proteins functional [18]. The activity of the M charged Z ions on the surface of the membrane and the cell wall {M^Z^}_0_ is described regarding the activity of the ions in the surrounding solution {M^Z^} and ψ_0_ using the equation of Nernst:

(1) $${\left\{M^{Z}\right\}}_{0}=\{M^{Z}\}\exp\left[\frac{-Z_{1}{F\psi }_{0}}{\mathrm{RT}}\right]$$

For example, a value of ψ_0_ = −59.2 mV will increase (compared to the contiguous solution) the activities of monovalent cations on the cell surface by 10 times, while for divalent and trivalent cations it will increase by 100 and 1000 times, respectively. The anions will show the opposite behavior. Thus, plant responses to ions, including intracellular functions such as electron transport in chloroplasts and mitochondria, will show little correlation with {M^Z^} and high correlation with {M^Z^}_0_ [18].

Changes in ψ_0_ are dynamic processes dependent on cellular metabolism and the composition of the cellular environment. The presence of cations reduces the negativity of ψ_0_, reducing the absorption and impact of other cations, especially those of toxic heavy metals, the order of effectiveness being: Al^3+^ > H^+^ > Cu^2+^ > Ca^2+^ ≈ Mg^2+^ > Na^+^ ≈ K^+^. The impact on anions is the opposite order. Although the protective effect of some cations such as Ca^2+^, Mg^2+^, and H^+^ has been interpreted as a result of the competition for ligands in the membranes, an alternative interpretation is that the impact of protective cations also comes from effects associated with surface charges and changes in ψ_0_ [44].

The changes in ψ_0_ impacts on the activity of specific receptors, channels, transporters, or in the dynamics of exosomes that are involved in the import of hormones and other growth regulators, the ions of essential elements such as NO_3_^−^, K^+^ and SO_4_^2−^, carbohydrates, lipids, lipoproteins, and the set of biomolecules necessary for growth and development [45,46]. Alterations in ψ_0_ will, therefore, have a significant effect on cellular metabolism.

The distribution of the charges on the surfaces of cell walls and cell membranes is heterogeneous at a scale of 10–50 nm due to the presence of proteins and other structures [20]. On the other hand, the distribution of the ions at the interface and in the solution with which the cell surface is in contact is considered as a product of thermal diffusion [18] (Figure 1). Therefore, the surrounding solution with ions in solution is a diffuse and homogeneous source of charges that interact with heterogeneous cell walls and membranes.

**Figure 3:**
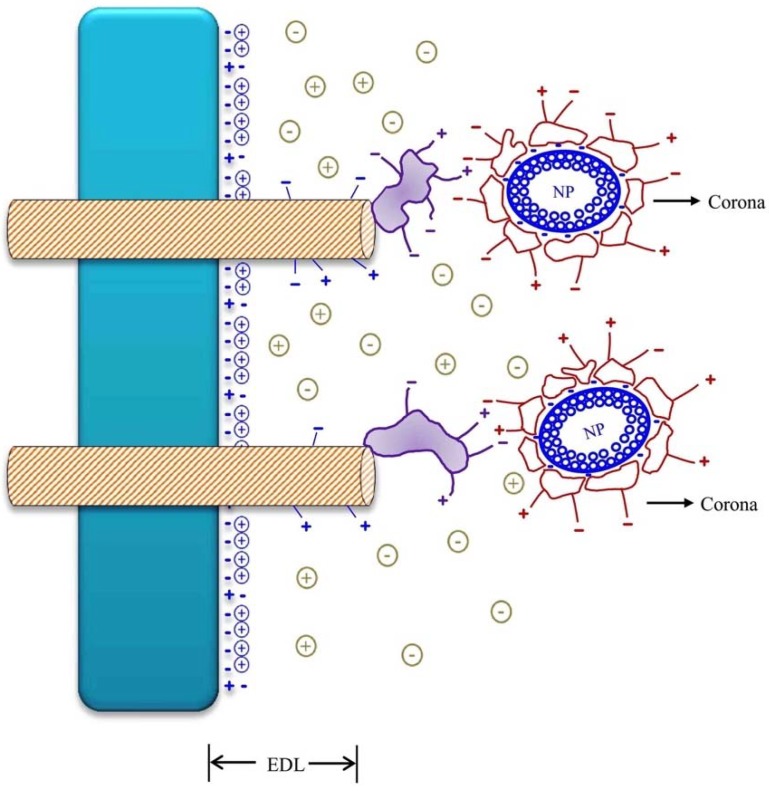
Graphical representation of the interaction of charges group of proteins of corona, cell wall or membrane.

NPs and NMs with a positive net surface charge or with exposed positively or negatively charged groups are able to interact with the negative surface charges of the wall and cell membranes, as well as with the external negative and positive charges of integral and peripheral proteins, without necessarily intervening a specific cellular receptor [44] (Figure 3). In the absence of a corona, the net surface charge of a pristine NP or NM is commonly negative, as is that of cell surfaces. The negative surfaces mean that the repulsion between the EDL of the cell walls and membranes and those of the pristine NPs and NMs would make the joining process between the two surfaces more difficult. In the absence of a corona, as in pristine NPs, the process would possibly depend on the particle´s hydrophobicity as an aggregation factor to decrease surface free energy in NPs and NMs [10,20].

Concerning the interaction with cell surfaces, NPs and NMs constitute a different case from that of ions [3]. It is assumed that the NPs and NMs are mobilized towards the cell surface by diffusion as it happens with the ions, but they do not seem to have specialized receptors. Therefore, the initial interaction with the cells seems to occur through attraction and complementation phenomena between the charged groups of the corona proteins and the peripheral and integral proteins that protrude from the EDL of the cell surfaces [21,47].

The temporal magnitude of the interactions of the charges depends on the stability of the NPs and NMs, related to the composition of the corona. This later constitutes the source of surface charges of an NP or NM. A first step of the cell–surface interaction NP occurs through the union of the groups of integral or peripheral proteins with the proteins of the corona, a union that probably occurs between groups with opposite charges. A second step of this interaction apparently occurs by the action of the free energy of the surface of the corona and its hydrophobicity. High free energy and hydrophobic groups tend spontaneously to decrease the surface exposure to water molecules of the apoplast and bind with cell surfaces [20].

The second step results in the union of the corona of the nanoparticle with the cell surface, extensively disturbing (a) the electrical potential of the surfaces of the membranes, (b) the transmembrane potential and (c) the activity of the ions associated with the Stern layer and the diffuse layer.

The changes in the electrical properties of cell surfaces occur because, unlike the ions in the EDL, the NPs and NMs are not diffuse and homogeneous sources of charges. Due to their surface energy and density, surface charges are heterogeneous sources with a high concentration of charges in a small volume. It is probable that, due to this last characteristic, the impact of the NPs and NMs on cellular metabolism will be different and higher than that exerted by the diffuse charges of ions in solution, which could partially explain the observation that small amounts of NPs or NMs exert significant effects on the plants [48].

The final result of the changes in the electrical properties of cell surfaces is the modification of the activity of integral proteins like receptors or channels that unchains metabolic adjustments and changes in the gene expression as the consequence of the perceived signals, which will give rise to a new cellular phenotype and a biostimulation response [13].

NPs and NMs are likely to induce biostimulation due to having a very high surface/volume value and a significant surface charge density. As non-diffuse source of charges, it can modify the electric and transmembrane potentials by the interaction with the surface charges of the cell wall and cell membranes. The strong surface interactions could explain the cellular impact of carbon NMs [29] and, in the case of metallic elements such as Cu, Fe, Ce, Ti, and Ag, the cellular responses so different induced by the ionic forms in comparison with the nanometric structures [13,16].

As an additional point, the high surface free energy of the NPs and NMs can induce, in later stages of the interaction, cellular signaling through mechanisms other than the surface charges of membranes and cell walls. It has been proposed that NPs and NMs and their aggregated forms can block the transit of ions and metabolites at cell walls and membranes [13,29]. Under conditions of high concentration of NPs this blockade would partially explain the toxicity of NPs [13]. Other mechanisms that possibly induce significant changes in cellular behavior would be the conformational changes induced by interactions between NMs and enzymes [11], and the mechanical stimulation of NP or NM [10,49], that could lead to a mechanoreceptor-dependent response located in cell walls or membranes. Indeed, the corona proteins, due to the surface free energy of NPs or NMs, can adopt abnormal folding states [20], which can trigger defense responses of the damage associated molecular patterns (DAMPs) type [5]. An example would be the unfolded protein response (UPR) pathway, which occurs in the endoplasmic reticulum but is activated by receptors located in cell membranes and activated in the presence of proteins damaged by salinity or high temperatures [50].

The result of the processes mentioned above would be the extensive modification of the activity of the receptor, transporter and signaling proteins of the membranes, with impacts on metabolism and gene expression. This physicochemical biostimulation phenomenon resulting from surface interactions is expected to occur with any type of NP or NM, regardless of their chemical composition. It is assumed that, under a specific range of concentrations, which depends on the particular type of NPs or NMs and their properties, the initial stimulus derived from the interaction of surface charges, triggers a positive response in the cellular metabolism or biostimulation.

The organic NPs with surfaces modified to have a cationic character like poly(amidoamine) dendrimers, show more significant interaction with cell surfaces than the anionic NPs [51], disturbing with greater intensity the ψ_0_ and causing signaling events that modify metabolism and gene expression. However, it should be noted that, despite their negative net charge, anionic NPs like cyanoacrylic NPs [51] also interact with membranes and cell walls, possibly through the charged groups of the integral and peripheral proteins of the membrane and by the participation of other properties of NPs such as hydrophobicity [26]. An example is the NPs of polystyrene, that shows a different impact on the cells according to the surface groups and charges, observing greater toxicity in those with superficial amino groups (with cationic character) in comparison with those containing carboxyl groups (with anionic character) or had unmodified surface [52]. Although the protein corona of the polystyrene NPs adopts a negative net charge, the most effective interaction with cell surfaces occurs through scavenger receptors that bind a variety of ligands when the pristine NP was of a cationic nature, whereas with the anionic pristine NPs the interaction occurs through specific native receptors [23,53]. In the polymeric NPs of chitosan, it has been found that the absorption rate and fate in the cell are related to the surface charge, with the anionic and neutral NPs with less cellular uptake being located in the lysosomes, while the cationic ones showed more quickly internalization with a lysosome and perinuclear location. The differential locations of chitosan NPs causes different cell responses [54]. In other NPs like dextran-coated superparamagnetic iron oxide nanoparticles differential cellular locations or impacts occur because the corona does not entirely cover its nucleus, leaving some functional groups exposed and active to interact with cell surfaces [39].

**Figure 4:**
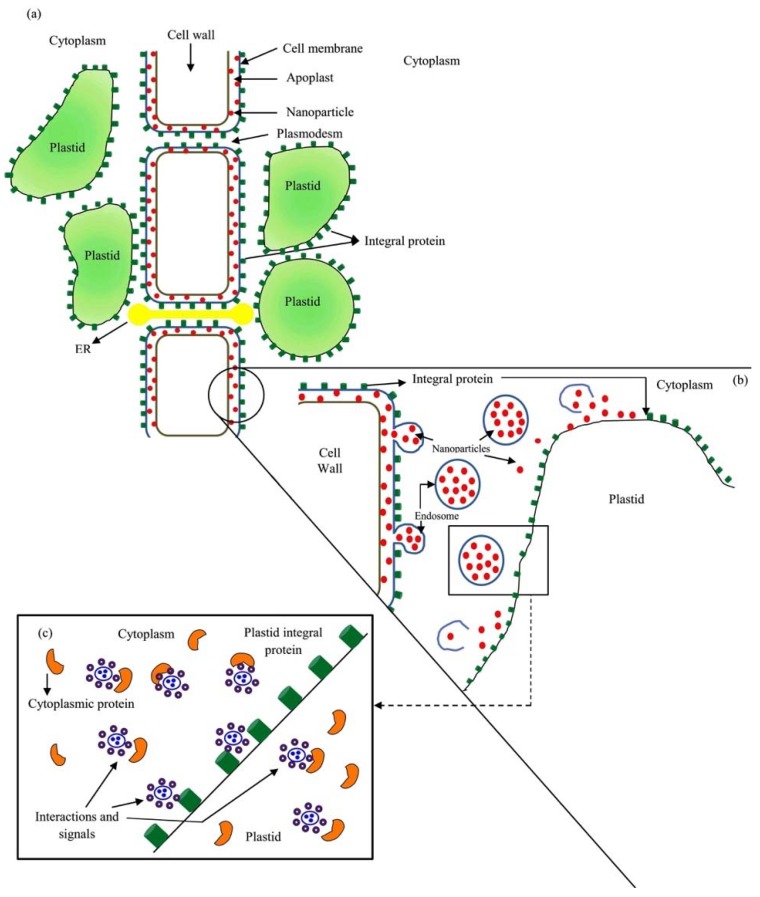
The interaction between nanoparticles or nanomaterials with cell surfaces. (**a**) Binding occurs to the cell wall or the membrane through to the free energy or charged groups of the surface of the corona surrounding the NP or NM, as well as to hydrophobicity phenomena that causes aggregation between surfaces. (**b**) Subsequently to the binding to the cell wall or membrane, the NP or NM is admitted to the cytoplasm due to direct internalization, endocytosis phenomena, or pore formation. (**c**) Once the NP is internalized to the cytoplasm, interactions with cytoplasmic proteins, internal membranes or organelles occur, which causes a series of metabolic adjustments and changes in gene expression that are signaled to other cells located in another tissues or organs of the plant. In this phase, it can happen that the interaction of the NP and the cellular medium release components (such as Cu^2+^ from a copper NP) that in turn trigger other cellular responses.

After the primary response to the changes in surface charges, a second series of biochemical stimuli are triggered by the entry of the NPs or by damage to the membranes or integral proteins that may occur in parallel with the internment. The primary and secondary responses will positively modify the cellular metabolism, causing biostimulation in the plant organism [14,55]. The process proposed above is schematized in the Figure 4.

In the metallic NPs, after the first surface interaction and cell signaling, the NPs internalized into the cell produces ionic forms of the element that are transported to the cytoplasm, impacting on metabolism according to concentration. This second-phase biochemical stimulation occurs by the presence of protein cofactors like Zn^2+^ or Cu^2+^ or by induction of oxidative stress [3,10,56,57].

Internalization occurs at the cell wall–apoplast or membrane–apoplast interface. Internalization occurs through pre-existing plasmodesmata pores up to 30 nm in diameter, modified pores with larger diameter, or by induction of new pores [58,59]. Intracellular mobility is achieved through the direct internalization or by endocytosis that occurs after the interactions between the surfaces of membranes and NPs; in the case of nanoporous materials, cell adhesion to the surface can occur [20,23,59,60,61]. Intracellular mobility has also been described for C nanotubes and other NMs [13]. The literature indicates that the NPs and NMs in the initial stages of internment do not maintain direct contact with the cytoplasm but remain in the endosomes. However, it has been described how endosome disruption events allow the release of NPs and NMs, thus reaching the cytoplasm, the internal membrane system and the organelle and nucleus membranes [62,63].

As with any other type of biostimulant, the definition of adequate concentration is specific for each type of NP or NM [16]. As expected, the increase in the concentration of the NPs or NMs beyond a certain threshold causes a negative response [7,13,63]. In the range of concentrations from very low to high, it is possible to observe positive responses followed by negative responses, and then followed by positive again. These alternate positive–negative responses may result in part from the cumulative dose over time, by phenomena of NPs aggregation, surface interactions, and cellular responses that do not follow a linear trend [3]. The absence of linear responses is explained by the presence of receptors that respond at different thresholds of the stimulus, as described for biostimulation with ultraviolet (UV) radiation [64]. The aggregation could be the explanation of the paradox that lower concentrations of NPs and NMs can induce more toxicity than higher concentrations [65]. Likewise, the non-linearity of the responses points to the need to distinguish the initial impact of the concentration of NPs and NMs (which we assume causes the first biostimulation response) from the subsequent cumulative impact over time that depends on the dose.

## 4. Responses of Plants to NPs and NMs

It is not the purpose of this manuscript to present a compilation of plant responses to NPs and NMs. The reader can refer to the works of [10,12,13,29,66,67,68], among others, to review the subject. Instead, the intention in this part of the manuscript is to present the information available in the literature about particular NPs or NMs. The purpose is to show that their use in specific concentrations induces, supposedly through interaction phenomena of charges between surfaces, changes in gene expression and positively impact on metabolism and growth.

As the first biostimulatory interaction between cells and NPs and NMs appears to be physicochemical, it is therefore independent of the chemical composition of the material and depends more on its surface properties. That is, it occurs whether the NPs or NMs are made up of elements essential for plant nutrition or if they include other elements or even engineered structures such as carbon nanotubes or graphene. This complex phenomenon has been extensively studied in the case of NPs and NMs for medical use [3], although there is less information published in the case of plant cells, where the presence of a cell wall imposes a fundamental difference concerning stimulus-response dynamics.

In the case of NPs of essential elements, the initial response of biostimulation is dependent on the interaction of the surfaces that occurs with concentrations lower than those necessary for the element to cover or complement the nutritional needs of the plant [7,69]. According to [3] it is possible that responses to NPs and NMs can occur even in nanomolar concentration ranges, whereas [70,71] described favorable responses of the plants to micromolar concentrations of Cu NPs applied in chitosan hydrogels.

In a second phase of the biostimulatory process, the NPs of essential elements would provide ions that, when incorporated into the metabolism, originate the classical nutritional responses described for this category of elements. By increasing concentration of NPs, as with any biostimulant, the response goes from positive to neutral or negative. On the other hand, in the segment of concentrations below those that induce a positive effect the response is generally neutral in the first phase of the interaction between NPs and NMs, so that it could be classified as a hormetic response [3,14]. This type of response was described in tomato using NiO NPs [63] and in rice applying ZnO NPs [72].

Almost all the literature about the impacts of NPs and NMs on plants refers to toxicity effects. Many studies were designed using concentrations of NPs and NMs greater than those used, for example, for trace elements such as Fe, Zn, Mn, and Cu (<3 mg L^−1^ or <0.05 mmol) in a nutrient solution. Another example would be graphene class materials, where concentrations of 500 to 2000 mg L^−1^ are toxic to plants [73], at 50–100 mg L^−1^ induces adaptation responses to stress as a higher concentration of the abscisic acid (ABA) hormone [74], whereas when applied to soil in low concentrations it promotes the absorption of water by the roots of plants [29,75]. However, the increasing agricultural use of NPs and NMs has added publications where the positive effects are explored using concentrations below those expected to be toxic [76]. This fact allowed to collect examples with NPs of TiO_2_ and Cu in which responses that can be described as biostimulation are indicated, possibly obtained through the action of mechanisms such as those described in the previous parts of this manuscript.

### 4.1. Nanoparticles of Titanium Dioxide (TiO_2_)

The nanomaterials of TiO_2_ have diverse uses such as solar cells, sensor devices, paints, and photo-catalysis. TiO_2_ exists in three forms: rutile (bulk), anatase and brookite. Most methods of synthesis and separation favor the more water-soluble and high surface-energy anatase structure over the lipophilic rutile structure [77]. The TiO_2_ NPs are among the most used in the industry, and it is estimated that 0.13 to 1200 µg of TiO_2_ NPs per kg of soil are incorporated each year [78]. Experimental tests to verify toxicity, transfer to ecosystems and their use as promoters of plant metabolism have also increased in number in recent years [79].

Ti is not an element considered essential for plants, but different reports point to it as a beneficial element for plants both when it is in soil and applied in foliar sprays [80]. In the soil, the Ti is relatively abundant and reaches 30 mg L^−1^ in the soil solution [78], so that the toxicity in the plants is reached with relatively high concentrations in the tissues, around 10^−4^ M [81].

Taking into account the high toxicity threshold of Ti on plants, the effects of TiO_2_ NPs on plants are perhaps mainly the result of surface interaction phenomena. The release of Ti^2+^, which could be beneficial for the plants, seems to be a mechanism that contributes less to the response considering that TiO_2_ is a poorly soluble compound in water [82]. The NPs of TiO_2_ have been applied as promoters of antioxidant activity and increased tolerance to different stresses: cold [83], heat [84], drought [80,85,86,87], NaCl [88], and ultraviolet (UV) light [89], in concentrations ranging from 5 to 2500 mg L^−1^ in plants of different species. Senescent seeds were treated with concentrations from 2500 to even 40,000 mg L^−1^, with the best results at 2500 mg L^−1^. In the latter case it is very likely that the TiO_2_ NPs have improved the germinative response through their capacity to increase the metabolism of the seed reserves and the transport of water to the internal tissues, as reported for the NPs of Ag [90] and graphene [91].

**Figure 5:**
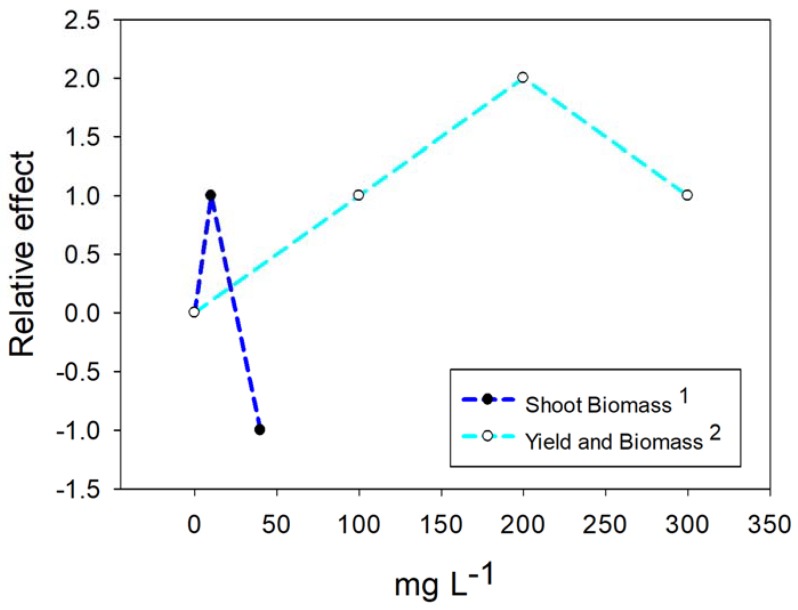
The relative effects of TiO_2_ NPs application on plants. On the Y axis the zero number represents the control treatment, a positive value represents a positive effect of the NPs, and a negative value represents an adverse effect on the specified variable. The superscript indicates the reference: dragonhead plants 1 [85], and wheat plants 2 [80].

In experiments without application of stress, 0.01 to 100 mg L^−1^ NPs of TiO_2_ were used, finding toxicity on flax plants with all concentrations [65]. Higher concentrations (from 300 to 2500 mg L^−1^) have been applied in spinach, benefiting the plants through the photocatalytic and photoprotective capacity of the TiO_2_ NPs [78,92,93]. In all the cases described, factors such as plant species, characteristics of the NPs and the conditions of the growth environment were decisive in the responses. However, a constant in all the works was that the positive responses were achieved with the lowest concentrations of TiO_2_. Figure 5 represents the results obtained on dragonhead and wheat plants by [85] and [80], respectively. It shows the response to low concentrations for the biomass variables of the plant, which constitute the physiological summary of the plant facing the stimulation of the NPs. It is possible to see that in both studies there is an intermediate concentration with the most significant positive impact, while the higher concentration resulted in lower biomass and yield.

Responses such as those presented in Figure 5, when not referring to essential elements, are considered as hormesis [14] and are characteristic of biostimulants that, according to their agricultural definition [4] induce greater growth without providing nutrients to the plants. Taking into account that the TiO_2_ NPs do not provide any nutrient, in addition to causing responses in plants from levels as low as 0.01 mg L^−1^, it can be assumed that they are acting in accordance with the accepted definition of biostimulant. The action would possibly occur through interaction between the surfaces of NPs and cells in a first phase, and later through aggregation phenomena that modify the response and decrease the toxicity, especially for high concentrations [3,65].

### 4.2. Nanoparticles of Copper (Cu)

Cu nanoparticles are used in industry for their catalytic, optical, electrical, and antimicrobial characteristics. In particular, this latter antimicrobial capacity makes Cu NPs, together with those of Ag, an attractive agent for use in clothing and footwear and textiles, food and water packaging and, in general, as a broad spectrum antimicrobial agent [94]. Unlike Ti, the toxicity threshold of Cu is reached at low concentrations. In a nutrient solution, the recommended amount of copper is 0.026 mg L^−1^, reaching toxic effects in a range of 0.06–0.32 mg L^−1^ depending on the plant species [95]. The Cu and CuO NPs in suspension release tiny amounts of Cu^2+^ ions (approximately 0.5%, equivalent to 5 mg L^−1^ of Cu^2+^ per 1000 mg L^−1^ of NPs of CuO) that are supposed to act in conjunction with the interactive effect of the surfaces of NPs and cells. The concentration threshold for adverse impact on plant metabolism appears located at 12–64 mg L^−1^ NPs of CuO. However, it was observed that even 10 mg L^−1^ of CuO NPs substantially decreased plant growth, showing significant DNA damage caused by the production of a significant amount of free radicals [96].

**Figure 6:**
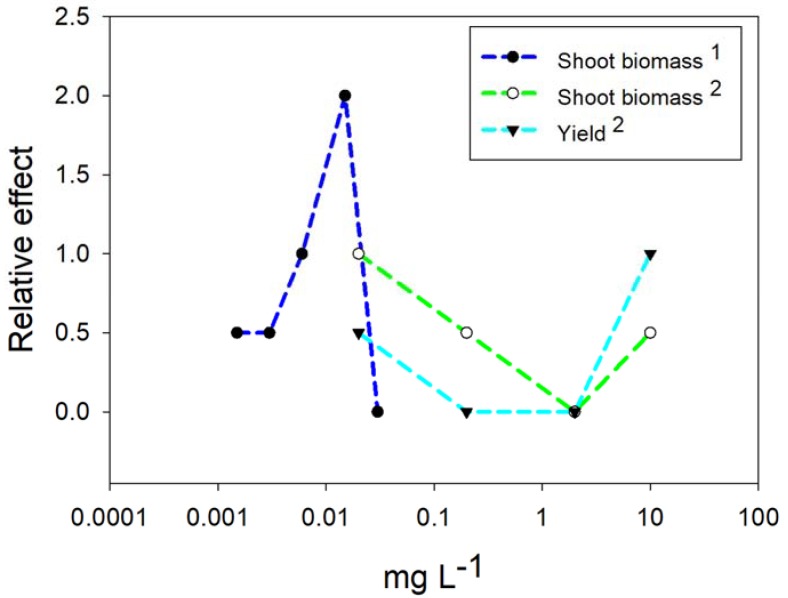
Relative effects of Cu NPs application on tomato plants. On the Y axis, zero represents the control treatment, a positive value represents a positive effect on characteristic specified. 1 [71]; 2 [70].

Foliar spraying is a practice of NPs and NMs application that substantially decreases the toxicity on plants thanks to the chemical interactions between the components of the leaf cuticle and the applied materials [97]. The NPs of Cu or CuO have been applied by foliar application with two localized applications using a suspension with 250 mg L^−1^ of Cu NPs to decrease the risk of toxicity [98]. This concentration of Cu NPs possibly would provide 1.25 mg L^−1^ of Cu^2+^. On the other hand, by applying CuO NPs (50, 100, and 200 mg L^−1^) by leaf spraying, mixed results were obtained, increasing the fruit production per plant, but decreasing some quality characteristics [99]. Another alternative is to apply to the substrate the Cu NPs in the form of complexes with biocompatible polymers, with positive effects at a concentration of 0.0015 mg L^−1^ up to 1 mg L^−1^ in tomato and *Vigna radiata* [71,100], equivalent to 0.02 to 10 mg of Cu NPs plant^−1^ in tomato plants [48,70]. Figure 6 shows the results obtained on the biomass and yield of the tomato plants when applying the substrate Cu NPs encapsulated in biocompatible polymers.

The definition of biostimulant does not include fertilizers with salts that directly contribute Cu^2+^ or Cu^+^ or other essential elements [4]. However, Cu or CuO NPs are not materials that directly contribute Cu and their application in the form of suspension releases approximately 0.5% of ionic Cu [96].

On the other hand, in Figure 6 the concentrations of Cu NPs added to the medium are very low, with positive responses of the plants being observed at levels, for example 0.01 mg L^−1^ of Cu NPs, with what would expect nanomolar concentrations of Cu^2+^, or approximately 500 times less ionic copper than the recommended for a nutrient solution. It is very likely that the biostimulation response observed is primarily due to the surface interaction phenomena of the NPs [3] followed by a possible effect of hormesis caused by the presence of Cu^2+^ in minimal amounts [14].

## 5. Conclusions

The surface charges of NPs and NMs interact with the surface charges of plant cells, inducing plant responses from biostimulation to toxicity.

When applied in small amounts by foliar spray or in nutrient solutions, NPs and NMs positively change the metabolism, composition and nutraceutical quality of food crops, as well as their tolerance to stress.

Biostimulation by NPs and NMs occurs in a specific range of concentrations that depends on the particular type of NPs or NMs and their properties. Biostimulation seems to occur in two stages. The initial phase, of a physicochemical nature, is the result of the interaction of surface charges; and the second phase results from the series of biochemical stimuli triggered by the entry of NPs and NMs or by the alterations in the membranes or integral proteins that can occur in parallel with the cellular internment.

Taking into account that the first phase of biostimulation seems to be physicochemical, it is concluded that virtually any NP or NM is capable of inducing biostimulation as long as it is applied in plants with the appropriate concentrations and physicochemical characteristics.
